# Clinical, Histopathological and Molecular Spectrum of Cutaneous Lesions in Myelodysplastic Syndrome and Myeloproliferative Neoplasms (MDS/MPN): An Integrative Review

**DOI:** 10.3390/cancers15245888

**Published:** 2023-12-18

**Authors:** Lucía Prieto-Torres, Luis Requena, Socorro Maria Rodríguez-Pinilla

**Affiliations:** 1 Department of Dermatology, Hospital Clínico Universitario Lozano Blesa, Universidad de Zaragoza, 50009 Zaragoza, Spain; 2 Department of Dermatology, Fundación Jiménez Díaz, Universidad Autónoma, 28040 Madrid, Spain; lrequena@fjd.es; 3 Department of Pathology, Fundación Jiménez Díaz, Universidad Autónoma, 50019 Zaragoza, Spain; smrodriguez@quironsalud.es; 4 Centro de Investigación Biomédica en Red de Cáncer (CIBERONC), 28040 Madrid, Spain

**Keywords:** myelodysplastic and myeloproliferative neoplasms, CMML, skin

## Abstract

**Simple Summary:**

Skin lesions in the context of MDS/MPN are frequent and poorly understood in the current literature. This paper thoroughly reviews the dermatoses reported so far in these patients based on their relationship with prognosis and its main clinical, histopathological and genetical features. Different histological patterns and mutations have been described in the literature. However, the true nature of the cells in these cutaneous infiltrates as well as their mutational background and the link with the underlying hematological neoplasm have to be further investigate in order to clarify their biological significance and the main therapeutic implications. A classification proposal is developed with the aim of helping dermatologists, hematologists and pathologists to recognize them when present in these complex patients. The figures and tables included in the text and in the supplemental material facilitate the understanding of each of the proposed categories. In addition, the main publications in which these dermatoses have been previously treated are indicated.

**Abstract:**

Myeloid neoplasms and acute leukemias include different entities that have been recently re-classified taking into account molecular and clinicopathological features. The myelodysplastic syndrome/myeloproliferative neoplasm (MDS/MPN) category comprises a heterogeneous group of hybrid neoplastic myeloid diseases characterized by the co-occurrence of clinical and pathological features of both myelodysplastic and myeloproliferative neoplasms. The most frequent entity in this category is chronic myelomonocytic leukemia (CMML) which is, after acute myeloid leukemia (AML), the main myeloid disorder prone to develop cutaneous manifestations. Skin lesions associated with myelodysplastic and myeloproliferative neoplasms include a broad clinical, histopathological and molecular spectrum of lesions, poorly understood and without a clear-cut classification in the current medical literature. The aim of this review is to describe and classify the main clinical, histopathological and molecular patterns of cutaneous lesions in the setting of MDS/MPN in order to improve the diagnostic skills of the dermatologists, hematologists and pathologists who deal with these patients.

## 1. Introduction

Myeloid neoplasms and acute leukemias include different entities that have been recently re-classified taking into account molecular and clinicopathological features. Two major articles were published in 2022, the ICC and the WHO classifications [1,2]. The myelodysplastic syndrome/myeloproliferative neoplasm (MDS/MPN) category comprises a heterogeneous group of hybrid neoplastic myeloid diseases characterized by the co-occurrence of clinical and pathological features of both myelodysplastic and myeloproliferative neoplasms. The most frequent entity in this category is chronic myelomonocytic leukemia (CMML) which is, after acute myeloid leukemia (AML), the main myeloid disorder prone to develop cutaneous manifestations [3]. Skin lesions associated with myelodysplastic and myeloproliferative neoplasms include a broad clinical and histopathological spectrum of lesions, which are poorly understood and without a clear-cut classification in the current medical literature. 

The aim of this review is to describe and classify the main clinical and histopathological patterns of cutaneous lesions in the setting of MDS/MPN in order to improve the diagnostic skills of the dermatologists, hematologists and pathologists who deal with these patients. Classically, these skin lesions have been divided into reactive or unspecific and neoplastic or specific ones. This traditional classification was based on the histopathological findings in cutaneous lesions [4]. However, within the past decade, the improvement in the molecular techniques to recognize gene mutations found in the cutaneous lesions of these patients has changed the previous concept of the reactive nature of some of these dermatoses [5]. The term “myelodysplasia cutis (MDS-cutis)” was coined to name some of the previously considered reactive neutrophilic infiltrates clearly related to MDS, in which the clonality of the myeloid infiltrate in the skin, common mutations in the cells of the cutaneous infiltrates and the hematological MDS/CMML clone found in blood and bone marrow (BM) and a close correlation between the course of the cutaneous lesions and the treatment and evolution of the disease, have been demonstrated. Some authors have adopted this term and also proposed the term CMML-cutis for the same scenario in CMML [6]. However, the literature is highly confusing with regard to the terminology used to name the dermatoses related to MDS/MPN, and the main goal of this review is to clarify these terms, fundamentally differentiating between two main groups of cutaneous lesions in these patients: dermatosis of indolent clinical outcome (see Table S1) and cutaneous processes associated with MDS/MPN and aggressive biological behavior (see Table S2). On one hand, MDS/MPN dermatosis of indolent clinical outcomes were previously defined as “non-specific”, but in which it has been currently shown that they share specific mutations with myeloid neoplastic cells in the bone marrow (BM). The fact that some of these MDS/MPN dermatoses only respond to the specific treatment of the myeloid neoplasm has led us to change the previous “non-specific” term to this new one. On the other hand, we define cutaneous processes associated with MDS/MPN and aggressive biological behavior as the ones with a clearly neoplastic histopathologic appearance, formerly described as “specific” which include the ones with the presence of blast leukemic cells in the cutaneous lesions. In addition, these cutaneous proliferations have been linked with a clearly worse prognosis. However, unlike the previous ones, skin lesions in dermatosis of indolent clinical outcome have not such a poor prognosis, and blast cells are usually not present in skin biopsies. This review, structured along clinical, histopathological and molecular lines, has allowed us to address conditions characterized by neutrophilic infiltrates, granulomatous infiltrates, mature plasmacytoid dendritic cell proliferations and other less frequently reported disorders in the group of indolent clinical outcome cutaneous infiltrates, and myeloid leukemia cutis or granulocytic sarcoma infiltrates, histiocytosis, histiocytic sarcoma and tumors of blastoid plasmacytoid dendritic cells in the group of processes associated with MDS/MPN and aggressive biological behavior. Finally, we include a small group of miscellaneous conditions less frequently reported in these patients, but with skin lesions which are worthy of mention, although they do not fulfill the criteria of either of the two previous major categories. All these cutaneous lesions demonstrate the plasticity of myeloid cells in the skin and BM, and their recognition can help pathologists, hematologists and dermatologists to better diagnose and manage these patients. 

## 2. Myelodysplastic/Myeloproliferative Related Dermatosis with Indolent Clinical Outcomes

Different histopathological patterns have been described in the classically considered reactive dermatoses in the setting of MDS/MPN. Since the description of shared molecular alterations, as well as a clinical course related to the hematological disease (persistence of skin lesions and response to the treatment of the myeloid neoplasm), a new term, myelodysplasia cutis (MDS-cutis), has emerged [7]. Below we will detail the main clinical, histopathological and molecular characteristics of these dermatoses, including forms with rapid response to oral corticosteroids and more persistent forms with shared molecular alterations with BM that could be discussed in what is described as MDS-cutis. To date, the biological meaning of the different clinical and histopathological variants of these dermatoses is unknown.

### 2.1. Neutrophilic Dermatoses

**Figure 1:**
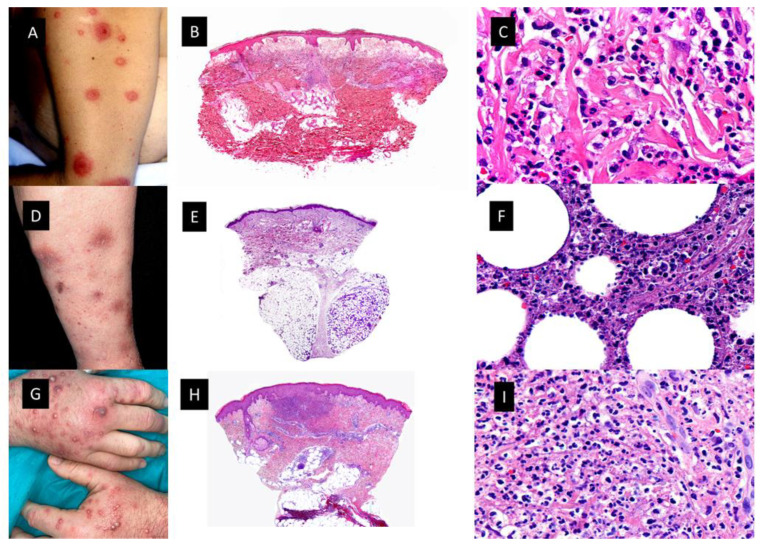
Neutrophilic dermatoses. (**A**). Classic Sweet syndrome (C-SS) presenting with edematous, erythematous and tender plaques located on the left arm. (**B**). At low magnification, intense edema of the papillary dermis and an underlying band-like, dense, dermal, inflammatory infiltrate mostly composed of mature neutrophils, with leukocytoclasis (HE staining 4×) (**C**). Detail of the neutrophils interstitially arranged in a linear way between collagen bundles (HE staining ×40) (**D**). Subcutaneous Sweet syndrome (S-SS) with erythematous and violaceous skin nodules located on the leg (**E**). Panoramic picture showing a mostly lobular subcutaneous infiltrate in the absence of vascular changes (HE staining 4×). (**F**). Higher magnification of the neutrophils located around fat lobules (HE staining 40×) (**G**). Vexas Syndrome with violaceous edematous papules and pustules with erythematous halo located on the dorsum of both hands (**H**). Dense dermal infiltrate located in superficial and middle dermis (HE staining 4×) (**I**). On higher magnification, dense infiltrate composed mainly of neutrophils (HE staining ×40).

Neutrophilic dermatoses (ND) (Figure 1) comprise a heterogeneous group of inflammatory skin disorders that share a similar histopathology, consisting of a sterile neutrophilic infiltrate. Clinical manifestations of the ND are diverse, even with variations in the same patient. Theoretically, the histopathologic location of the neutrophilic infiltrate (epidermis, dermis, subcutis), the disease course and the clinical appearance help to make the differential diagnosis among different ND, such as Sweet syndrome (SS), pyoderma gangrenosum (PG), Behçet syndrome (BS), erythema elevatum diutinum (EED) and neutrophilic eccrine hidradenitis (NEH) [8]. 

#### 2.1.1. Sweet Syndrome

In 1964, Robert Douglas Sweet reported eight patients with an “acute febrile neutrophilic dermatosis” that afterwards was renamed SS [9]. Clinically, patients presented with fever and edematous, erythematous tender cutaneous plaques located on any area of the skin surface, but mainly involving the upper trunk. The usual histopathological findings consisted of intense edema of the papillary dermis and an underlying band-like, dense, dermal, inflammatory infiltrate mostly composed of mature neutrophils, with leukocytoclasis but without features of vasculitis. (Figure 1B,C) In rare instances, however, typical lesions of SS may show histopathological features of leukocytoclastic vasculitis, which seems to be a secondary epiphenomenon. Approximately 21% of patients with SS have an associated malignancy [10]. Malignancy-associated SS is more commonly reported with hematological malignancies and MDS/MPN compared to solid-organ malignancies. The most common associations are AML followed by MDS. SS may precede or follow a diagnosis of malignancy, and it has even been described as a signal of cancer recurrence [11]. There are several clinical and histopathological variants of SS, including localized SS, histiocytoid SS (H-SS), subcutaneous SS, acute necrotizing SS and xanthomatized neutrophilic dermatosis. One clinicopathological variant that deserves special attention in patients with myeloid neoplasms is H-SS. 

##### Histiocytoid SS (H-SS)

**Figure 2:**
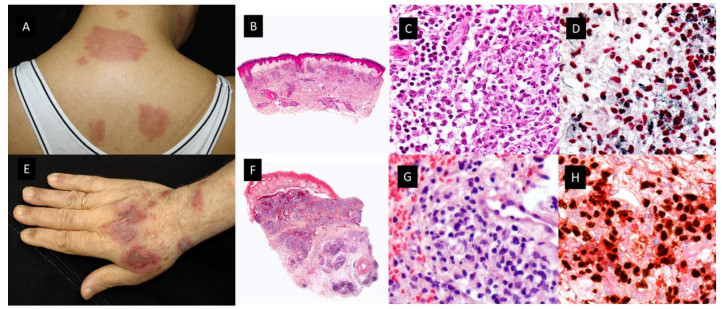
Clinicopathological features of Histiocytoid Sweet syndrome(H-SS) and Myelodysplasia cutis (MDS-cutis). (**A**) H-SS clinical picture showing erythematous and edematous plaques on the back and posterior region of the neck (**B**) Scanning power shows edema and nodular infiltrates in the superficial dermis. (HE staining 4×). (**C**) The dermal nodules are mostly composed of mononuclear cells with twisted vesicular nuclei and scant eosinophilic cytoplasm. (**D**) Double immunostained specimen with nuclear myeloid nuclear differentiation antigen [MNDA, black] and cytoplasmic myeloperoxidase [MPO, red]) (40×). (**E**) MDS-cutis showing edematous plaques on the dorsum of the hand and arm, with raised erythematous border and a slightly depressed violaceous center. (**F**) Histopathologic features consist of edema in the papillary dermis and multiple nodular infiltrates in the superficial and deep dermis; there are also perivascular infiltrates around deep dermal vascular plexus (HE 4×). (**G**) On higher magnification, atypical, dermal hematolymphoid infiltrate and some red cells, the atypical cells demonstrate reniform nuclei and eosinophilic cytoplasm and include some pseudo-Pelger–Huet anomaly (HE 40×). (**H**) Double immunostained specimen with myeloid nuclear differentiation antigen [MNDA, black] and cytoplasmic CD123 [CD123, red].

This histopathologic variant of SS, first described by Requena et al. in 2005 [12], is characterized by a dermal infiltrate of mononuclear cells of histiocytic appearance, but strongly positive for myelocytic and promyelocytic markers, as demonstrated by the double immunostain for myeloperoxidase (MPO) and myeloid cell nuclear differentiation antigen (MNDA) [13]. As in classic SS, patients with H-SS present with tender erythematous plaques and nodules on the extremities and trunk accompanied by systemic symptoms such as fever and arthralgia [12,13]. (Figure 2A–D) Association with malignancy ranges from 30% to 61% and the most frequent associated neoplasia is MDS.

In the first 41-patient-series published by Requena et al., there were 7 patients with associated malignancies, including CMML (case 3) “lymphoma” (no more specific diagnosis could be obtained) (case 21), monoclonal gammopathy of undetermined significance (case 24), renal carcinoma (case 29), breast carcinoma (case 28), chronic B lymphocytic leukemia (case 38), and multiple myeloma (case 39) [12]. In a series of nine patients, Vignon-Pennamen et al. suggested for the first time that this histopathological variant could be more frequently associated with AML and MDS than classic SS [14]. Later, in 2015, Ghoufi et al. published a larger series, which included 62 patients with a histopathologic diagnosis of SS, 22 cases with H-SS and 40 cases with the classical neutrophilic variant (N-SS) [15]. They found that MDS was diagnosed in 7 of 22 patients with H-SS and only in 1 of 40 patients with N-SS (*p* < 0.001). In three H-SS patients with MDS, the cutaneous lesions preceded the hematological diagnosis by more than 6 months. Therefore, they proposed that a complete hematological assessment and close follow-up should be mandatory in patients with H-SS [15]. In contrast, Alegría et al. found that H-SS was not more frequently related to hematologic malignancies than N-SS [13]. In their series of 33 patients with H-SS, 8 suffered from hematological malignancies, including 3 with MDS and 2 with CMML. In these patients, a CD163+/MPO+ population was absent in the infiltrate, whereas double immunostain with MNDA/MPO was intensely positive in most cells of the infiltrate, whereas a minor proportion of cells expressed the double immunostain for MNDA/CD163. The skin lesions appeared 5 and 2 years before the diagnosis of the hematological condition [13]. 

The initial presumption of the reactive nature of SS, including variants like H-SS, was supported by the acute onset of the skin lesions and the complete response to steroid therapy. However, the improvement in molecular techniques has demonstrated clonally related cells in both the skin and bone marrow of hematological patients with N-SS and H-SS [5]. In 2020, a molecular study including 10 patients with SS and AML, myelodysplastic syndrome, myeloproliferative neoplasm or MDS/MPN was published. Eight patients had N-SS and 2 H-SS. They performed Next Generation Sequencing (NGS) analysis of paired skin-lesion biopsies and detected 35 out of the 37 mutations found in hematopoietic samples, without additional mutations. Seven patients exhibited identical mutational profiles in both tissues. In all mutated cases (n 9), mutations of the major clone of the myeloid neoplasm were also found in the cells of cutaneous infiltrates. CD34 and myeloperoxidase immunohistochemical staining of skin biopsies revealed very few immature myeloid cells in only 2 out of 10 patients, ruling out leukemia cutis. Most mutations detected in skin-lesion samples were at relatively high allelic burden (median 20%, at least one mutation with variant allele frequencies of 10% for each patient) indicating that they did not relate to rare infiltrating blast cells or blood contamination of skin biopsies. The authors assumed that the process of migration and infiltration of the skin in SS may be related to the tumoral phenotype of myeloid cells, rather than only triggered by cell extrinsic factors [5]. 

These NGS studies in the skin of patients with MDS and ND, especially H-SS, also support the concept of MDS-cutis [16]. 

##### Other Morphological Variants of the Sweet Syndrome

There have been other clinicopathogical variants reported in the literature in the setting of MDS/MPN mentioned above [17]. Among all of these variants, it is worth highlighting the subcutaneous Sweet syndrome (S-SS) or neutrophilic lobular panniculitis (Figure 1D–F) [18,19]. Other names employed in the literature include “Sweet’s panniculitis”, “Sweet´s-like neutrophilic panniculitis” and “neutrophilic panniculitis associated with myeloid disorders”. Primary neutrophilic panniculitis, defined as neutrophilic infiltrates confined to the fat lobules of the subcutis as opposed to extension of a dermal neutrophilic infiltrate into the subcutis (secondary neutrophilic panniculitis) has been reported in the literature as a histological pattern seen in a heterogeneous group of disorders including infectious panniculitis, “reactive panniculitis reaction”, which is associated with some bacterial antigens and usually has vascular changes, and leukemia cutis [20]. Some of these diseases may be readily diagnosed, but others are less distinct clinically and histopathologically. For example, it may be difficult to distinguish between S-SS and infectious panniculitis [20]. 

S-SS in the setting of myeloid disorders has been described in scattered case reports and small series of cases as erythematous skin nodule(s) generally located in the extremities with the trunk and the face affected less frequently. Cutaneous lesions are usually accompanied by fever and they resolve within days or a few weeks of oral steroid treatment like classic SS (C-SS). Histopathologically, these nodules are characterized by a dense neutrophilic infiltrate confined to the subcutis in the absence of vascular changes. Unlike C-SS, papillary dermal edema is minimal. Both septal and lobular panniculitic patterns have been reported, the lobular one being the commonest form. Interestingly, occasional large epithelioid cells consistent with reactive stromal fibroblasts and abnormal nuclear segmentation (hyposegmentation or hypersegmentation) of neutrophils have been described in these myeloid-related cases [20]. AML and MDS are the myeloid neoplasms more frequently related to S-SS in the reported cases [20]. A variant of subcutaneous H-SS has also been reported heralding transformation of MDS into AML [21] in a relapsed myeloblastic leukemia [22] and in MDS-refractory anemia [23].

#### 2.1.2. Vexas Syndrome

In 2020, a new adult-onset autoinflammatory syndrome with overlapping features with MDS or multiple myeloma (MM) and inflammatory diseases such as relapsing polychondritis, SS, polyarteritis nodosa or giant-cell arteritis was described in 25 men with somatic mutations in p.Met41 of *UBA1*, the major E1 enzyme that initiates ubiquitylation [24]. This enzyme is encoded by the gene *UBA1* located on the X chromosome. Its estimated prevalence is about 1 in every 13591 adults [25]. 

The acronym, VEXAS, stands for *v*acuoles, *E*1 enzyme, *X*-linked, *a*utoinflammatory and *s*omatic syndrome. Somatic mutations, first acquired and then clonally selected, have been implicated as previously referred to in neoplastic hematological diseases [26]. With VEXAS syndrome, the authors of the original description used a genotype-driven approach to identify a genetic cause of an autoinflammatory disease. All patients with *UBA1* mosaic mutations had predominantly wild-type lymphocytes (T and B cells) and mutant myeloid cells (neutrophils and monocytes) in peripheral blood, although a patient with vacuolated lymphoid precursors was also reported [27]. Hematopoietic stem cells and multipotent progenitors isolated from BM had abundant mutant cells. The patients began to develop the inflammatory syndromes in the fifth to seventh decade of life. Patients had progressive hematological abnormalities, including macrocytic anemia, thrombocytopenia and myeloid dyspoiesis. Co-occurrence of VEXAS with hematologic neoplasms has been described with MDS and plasma cell dyscrasia, and recently a male patient with chronic myeloid leukemia (CML) and VEXAS [28] has also been reported. The dermatological manifestations include neutrophilic dermatoses similar to SS with a poor response to conventional therapies and frequent relapses, similar to the previously described MDS-cutis patients [29]. 

#### 2.1.3. Myelodysplasia Cutis

Osio et al. identified in a series of 150 patients with MDS and cutaneous lesions 24 patients with non-blastic tumor cells in the dermal infiltrate, defined as medium-sized immature myeloid cells with abundant eosinophilic cytoplasm and twisted nuclei or the pseudo-Pelger–Huet anomaly (see Figure 2E–H) [30]. They found that these cells had a combined myeloid and monocytic immunophenotype, with the expression of both MPO and CD163 or CD68 antigens and negativity for CD34, CD56 or CD117. The proliferative index was low in 56% of cases (<10% of positive Mib-1 cells) or intermediate in 44% of cases (10 to 66% positive Mib-1 cells). In addition, the cutaneous infiltrate was rich in mature neutrophils and normal CD3+ T-lymphocytes. The presence of edema in the superficial dermis was a frequent finding (67% of samples) [30]. Fluorescent in situ hybridization (FISH) analyses showed common cytogenetic abnormalities in the skin and the BM of 4/6 patients. Previously, only Sujobert et al. had demonstrated a clonal relationship of this kind in neutrophilic dermatoses associated with AML [31]. These authors postulated that some cases previously reported as “H-SS” in the course of MDS, especially those with a poor response to treatments such as hydroxychloroquine, dapsone, colchicine or thalidomide or patients with steroid dependence or in need of high dose oral prednisone could have MDS-cutis. Furthermore, they compared the survival rate and other clinicopathologic characteristics of patients with MDS-cutis and leukemia cutis, and they found significant differences between the two groups [30]. Regarding overall survival (OS) after the skin diagnosis, it was significantly longer in MDS-cutis patients than in patients who suffered from leukemia cutis (62 vs. 5 months, (*p* < 0.001)). In addition, there were other discriminant histopathological features related to survival influence, such as positivity for CD34, CD56 or CD117 for shorter survival in patients with leukemia cutis (*p* < 0.059), and the presence of CD3+ T lymphocytes (*p* < 0.001), edema (*p* < 0.01) and a lower Mib-1 proliferative index (*p* < 0.05) for longer survival in patients with MDS-cutis. Clinically, leukemia cutis patients usually have persistent skin nodules, whereas in MDS-cutis patients there are flares and relapses of cutaneous lesions. These lesions were nodules in leukemia cutis (*p* < 0.01) and erythematous plaques (*p* < 0.001), with a frequent annular pattern (*p* < 0.05) accompanied by fever, or arthralgia (*p* < 0.01) in MDS-cutis patients [30]. 

Finally, there has been a recent report of seven patients with MDS-cutis, one of them with *UBA1* mutation (VEXAS syndrome), in which NGS analysis found one to five mutated genes both in the BM (with a median VAF of 30%) and in the cutaneous lesions of the same patients (with a median VAF of 11%) [16]. In addition, there were some mutations detected in one tissue but not in the other, supporting the possibility of clonal evolution in one of the tissues [16]. They suggested that MDS-cutis should be considered as differential diagnosis of leukemia cutis, classical SS and MDS-associated vasculitis. MDS-cutis may potentially be underdiagnosed if a molecular investigation is not performed, especially in patients with H-SS. Unlike what happens in patients with N-SS, MDS-cutis patients seem to be more resistant to steroid therapy, and they may respond to hypomethylating agents, like Azacytidine, although this sensitivity still must be confirmed in larger prospective series [16]. 

### 2.2. Granulomatous Dermatoses 

Interstitial granulomatous dermatitis (IGD) was first described by Gottlieb et al. in 1995 as a granulomatous dermatitis with interstitially disposed dermal infiltrates composed mainly of histiocytes in a palisaded arrangement around the foci of degenerated collagen bundles, with scattered neutrophilis and eosinophilis [32]. Shortly after, Chu et al. proposed the term “palisaded neutrophilic and granulomatous dermatitis (PNGD)” for the papular eruption on the extensor surface of extremities described in the context of collagen vascular diseases [33]. Both terms, IGD and PNGD, as well as the overlapping between IGD and granuloma annulare, are now included by some authors in the spectrum of the so-called reactive granulomatous dermatitis [34]. 

These cutaneous lesions have been mainly related to rheumatologic diseases and drug reactions. However, the association of PNGD or IGD with hematological malignancies, including CMML and MDS, has been reported firstly as a reactive dermatitis in the setting of the hematological condition and lately as a possible skin manifestation of the disease [35,36]. 

**Figure 3:**
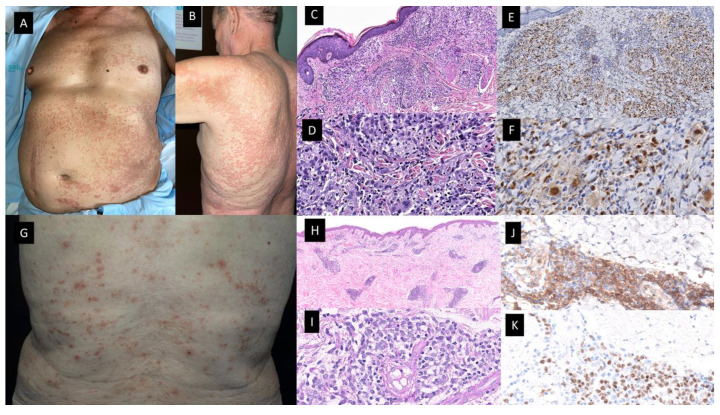
Granulomatous dermatitis (**A**–**F**) and mature plasmacytoid dendritic cell dermatosis (**G**–**K**). Patient with a diagnosis of CMML presented with multiple 1–2 mm erythematous-brownish papules that coalesce on (**A**). His anterior trunk and abdomen as well as on (**B**) Back and upper arm and extremities (not shown). (**C**,**D**) Hematoxylin-eosin-stained specimen. (**C**) Histopathological features consist of a dermal inflammatory infiltrate composed of epithelioid histiocytes, giant cells and lymphocytes, with rare neutrophils, configuring ill-defined granulomas (20×) (**D**) On higher magnification, the detail of the granulomas with multinucleated giant cells (40×). (**E**,**F**) On immunohistochemical study, CD68 stained the histiocytes [(**E**) (20×), (**F**) (40×)]. (**G**) Multiple erythematous and purpuric papules on the back. (**H**,**I**) Hematoxylin-eosin-stained specimen. (**H**) Dermal nodular infiltrate with perivascular and periadnexal arrangement (20×) (**I**). Detail of lymphocytic infiltration as well as interspersed larger, paler cells corresponding to mature PDC (40×). (**J**) Cytoplasmic CD123 immunostaining highlighting clusters of plasmacytoid dendritic cells (PDC) (40×) (**K**) Nuclear SPIB stain in PDC (40×).

Federmann et al. published in 2017 the findings regarding three patients with widespread papular eruptions resistant to conventional skin-directed therapies, which histopathologically showed interstitial granulomas composed of epithelioid histiocytes in the reticular dermis arranged around the foci of degenerated collagen and variable neutrophilic inflammation consistent with PNGD (see Figure 3A–F) [37]. The granulomas showed a similar immunohistochemical profile, with positivity for CD14, CD68 (PG-M1) and CD123, whereas only very few cells were positive for TCL1 and CD303. CD56, S100 protein and CD1a were negative. The most interesting contribution of this study was the finding of SRSF2 P95 hotspot mutations, found in 40–50% of patients suffering from CMML, which were retrospectively detected in both the skin and BM biopsies of the three patients. In addition, in one of them, these mutations were already found 5 years before the diagnosis of the hematologic neoplasia [37]. Since then, only a few more cases have been reported [36,38,39]. All but one were patients with CMML, in all cases where molecular techniques were performed, SRSF2 mutations were found in the BM and in the cutaneous infiltrates of the patients. For that reason, Enescu et al. proposed that this kind of dermatitis is specific for CMML patients with this molecular alteration [39]. The molecular technique used for the study of mutations in the BM and skin of these patients was variable, including NGS, pyrosequencing or RFLP analysis by BsaJI digestion and/or sequencing of SRSF2 polymerase chain reaction products containing the hotspot region in codon 95 (2 p.Pro95His;1 p.Pro95Leu) [36,37,39].

Before the description of the Federmann cases in 2017, this type of granulomatous reaction had been published in association with MDS, and the authors argued that it could be a manifestation of the disease, because the cutaneous lesions did not respond to conventional treatments for reactive dermatoses, but they improved with the specific chemotherapy treatment for the myeloid neoplasia [35]. 

### 2.3. Mature Plasmacytoid Dendritic Cell Dermatoses

Plasmacytoid dendritic cells (PDC) represent a subset of cells within the immune system with distinctive morphology, immunophenotype and which develop specific functions [40]. Within the bone marrow, the total representation of mature plasmacytoid dendritic cells is less than 1% of the total nucleated cells. There are some inflammatory diseases in which an increased number of PDC has been described. They include Castleman disease, lupus erythematosus and Kikuchi-Fujimoto lymphadenopathy [41]. In addition, it is already known that the number of PDC increases in both CMML and MDS BM biopsies [40,42,43]. The presence of CD123-positive monocyte nodules had been reported only in CMML, whereas they were not in BM samples from CML and atypical CML. Besides, their amount has been related to prognosis in previous publications [44]. To date, two extremely rare PDC neoplasms have been diagnosed in patients with other hematological diseases: on the one hand a very aggressive one, blastic plasmacytoid dendritic cells neoplasms (BPDCN) and on the other hand a more indolent one consisting of mature PDC proliferation. 

In 2012, Vitte et al. published an article describing a series of 42 patients with CMML and skin infiltrates [45]. They classified them in four different groups depending on the nature of the skin cells: (1) skin tumors composed of myelomonocytic cells; (2) skin tumors of mature PDC; (3) cutaneous blastic PDC tumors (BPDCT); and finally (4) skin tumors formed by blastic indeterminate dendritic cells. Their study included 16 patients with tumors of mature PDC. These patients were 15 males and only 1 female, whose median age at presentation was 76,35 years. Clinical features consisted of erythematous and itching maculo-papules. Generally, cutaneous lesions appeared concomitantly with the diagnosis of CMML or after a period of time. There are some other myeloid neoplasms that have been related to both mature PDC proliferations and BPDCN in previous literature which are MDS and acute leukemia with monocytic differentiation [39,41,42,43].

In 2022, Machan et al. published the findings regarding six patients with pruritic papular eruptions histopathologically characterized by dermal infiltrates of mature T-lymphocytes with large clusters of CD123+ and SPIB+ cells and with a lack of myeloid cell nuclear differentiation antigen (MNDA). (See Figure 3G–K) These cells were also negative for CD4, myeloperoxidase, TDT, CD56, BCL2, and cytotoxic markers. Neither CD117 nor CD34 positive cells were present on the infiltrates. It should be noted that immunostaining with S-100 protein and CD1a highlighted an increased number of S-100 protein and CD1a positive cells, which were mainly localized in the epidermis and superficial dermis. However, they found only scattered double positive cells for both S-100 CD1a markers. Four patients have been diagnosed with CMML and two had MDS (AREB-I and MDS with 5q deletion). The skin lesions developed in all cases coincidentally with either progression or full-establishment of their hematological disease [46]. Clinically, the skin lesions were more heterogeneous than in the case of the granulomatous dermatoses previously described, and unlike the previous ones, most cutaneous lesions disappeared spontaneously or after oral or topical corticosteroid treatment. One patient presented with multiple erythematous macules and papules in the same stage of development, which were spread on the trunk and extremities with sporadic paler halos, and a clinical diagnosis of drug eruption versus vasculitis was established. Another patient presented with isolated erythematous nodules located on both the left flank and breast, which were clinically interpreted as suggestive of primary cutaneous lymphoma. Finally, small papules on the extremities mimicking insect bites were also described. Biopsy samples in these patients showed focal epidermotropism in three patients, with vacuolar degeneration of the dermo–epidermal interface and folliculotropism of the infiltrate, but without mucin deposition. If we compare histopathologically the Vitte et al. reported cases with the cases of Machan et al., clusters of mature PDC, lymphocytes, plasma cells and indeterminate dendritic cells were present in the dermis in a similar proportion in both series [44,45].

This histopathology with features mimicking lupus erythematosus was previously reported in a patient with CMML type 2 diagnosed simultaneously with the appearance of the cutaneous lesions [47]. In this study, PDC aggregates were positive for CD4, CD68, granzyme B, CD123, BDCA-2/CD303, TCL1 and the IFN-α inducible protein, myxovirus A (MxA). In addition, PDC were aberrantly positive for CD5 and CD7, two antigens usually not found on normal PDC. CD56 staining was negative. They did not find an abnormal number of Langerhans and dermal dendritic cells expressing CD1a, S100 protein or langerin/CD207. Besides, the patient shared the same chromosomal abnormality (chromosome 13 trisomy) detected by Fluorescence in situ hybridization (FISH) analysis in his cutaneous lesions and BM. The cutaneous lesions resolved with chemotherapy for the hematological neoplasm and the patient died one year later after progression to AML without the recurrence of the cutaneous rash [46]. 

From a molecular point of view, in the Machan et al. series, patient number 2 had mutations in *TET2*, *SRSF2* and *ASXL1* genes in both BM and skin. They performed NGS techniques and found an allele frequency of *TET2* gene mutations of 90.3% and 15.4% in BM and skin, respectively; 45.8% and 7.3% for *SRSF2* gene in BM and skin, respectively; and 39.9% and 5.9% of the *ASXL1* gene in either BM or skin. The combination of *TET2*, *SRSF2* and *ASXL1* gene mutations is highly specific (98%) for the diagnosis of CMML and has proven to have prognostic implications [48]. Furthermore, the BM of their number 3 patient, who had a diagnosis of AREB-I, showed mutations of genes *DNMT3A* and *IDH1* with an allele frequency of 38.5% and 32.9%, respectively, via NGS. They also performed pyrosequencing of the *IDH1* gene on paraffin-embedded tissue from cutaneous lesions of this patient and discovered the same mutation [46]. It is worth mentioning that both *TET2* and *DNMT3A* gene mutations have been related to clonal hematopoiesis of indeterminate potential (CHIP) in myeloid neoplasms [48], and also that *IDH1* mutations have been related to a poor outcome in MDS [49]. 

Finally, regarding the evolution and follow-up of the patients, cutaneous lesions of the Vitte et al. series either did not respond to treatment in 12.5%, and 37.5% of the patients showed a partial response, whereas in the Machan et al. series, all skin lesions resolved spontaneously or after topical or oral treatment with steroids. In addition, in all cases published by Machan et al., the skin lesions developed coincidentally with the progression of the full-establishment of their hematological condition. Despite this, in the study published by Vitte et al., the group of patients with mature PDC proliferations seemed to have better prognoses compared to the other ones, which were more consistent with leukemia cutis (see below) [45].

### 2.4. Other Myelodysplastic/Myeloproliferative Dermatosis

To conclude the discussion about MDS/MPN-related dermatoses with an indolent clinical course, it is necessary to mention a rare clinical form of MLC that can be seen in CMML patients. It has been described as chilblain-like eruptions that can be the first manifestation of a blast crisis or the relapse of AL being an important clue for the diagnosis [50] of chronic myelocytic leukemia [51]. Skin lesions consist of bluish erythematous macules with mild itching on the toes of both feet. The immunohistochemistry of the infiltrating cells that were present in cutaneous lesions showed positivity for CD68 and were partially positive for CD14. These cells were negative for CD13, CD33, CD34 and CD56 [51]. Similar waxing and waning chilblain-like lesions of 8 months’ duration were described in a 6-year-old girl diagnosed with juvenile myelomonocytic leukemia. Her skin lesions were histopathologically characterized by a dense superficial perivascular dermal infiltrate, which was CD3-, CD43+ and lysozyme +, suggesting a monocytic origin [52]. 

## 3. Cutaneous Processes Related to Either MDS/MNP and Aggressive Clinical Behavior

Disease progression is a major source of mortality in myeloid neoplasms, including MDS/MPN. Apart from transformation to AML and overgrowth of pre-existing hematopoiesis by the myeloid neoplasm without an additional transforming event, there are other recurrent less well-known scenarios that exhibit a propensity for extramedullary sites like the skin. These scenarios include: (1) acquisition of MDS features in MPN, (2) acquisition of MPN features in MDS, (3) progressive myelofibrosis (MF), (4) acquisition of CMML-like features, (5) development of granulocytic sarcoma/leukemia cutis, (6) lymphoblastic transformation, and (7) histiocytic and dendritic cell overgrowth [53]. Here, we are going to describe the cutaneous lesions more frequently found in MDS/MPN neoplasms with disease progression and how the gain of distinct mutations/mutational patterns seem to be responsible or at least concomitant with these clinical scenarios. 

### 3.1. Leukemia Cutis/Myeloid Leukemia Cutis

Leukemia-specific skin infiltrations are often named leukemia cutis (LC) in the literature, which includes both lymphoid and myeloid leukemias. In the case of myeloid disorders (including AMML, CMML, myelodysplasias, myeloproliferative syndromes such as polycythemia vera, essential thrombocythemia and idiopathic myelofibrosis), myeloid leukemia cutis (MLC) seems to be a more appropriate term. Other names used to designate MLC are chloroma, myeloid sarcoma and granulocytic sarcoma, which all mainly signify extramedullary localization of a myelomonocytic malignancy [54]. Most of these myeloid disorders represent AMLs, particularly those of monocytic differentiation. However, other myeloid disorders, less frequently represented as CMML, refractory anemia (RA), and MPS, may also involve the skin at the time of their blastic transformation. The frequency of LC is reported at 2.1–30% depending on the underlying form of leukemia [4] and it can reach 50% in some series of the AML of monocytic subtypes [55,56]. Regardless of the type of myelogenous leukemia, the onset of specific skin manifestations clearly correlates with an aggressive course and short survival [56]. 

#### 3.1.1. Granulocytic Sarcoma 

In 2011, Mathew et al. performed a retrospective analysis exploring the role of LC in the disease progression of CMML [57]. Of 108 patients in their series with CMML, 11 patients (10.2%) had LC including 1 patient with multicentric reticulohistiocytosis (MCRH) and 1 patient with BPDCN, which they define as its equivalent (2 patients). Four of these patients developed AML within 0–4 months. The remaining seven patients demonstrated increased monocytes (<20% blasts), with three of them developing extramedullary involvement. They also showed that the overall survival (OS) from MLC to disease progression was 7.8 months. In addition, the OS from diagnosis to the last follow-up in patients with MLC was 28.2 months, shorter than patients without MLC (44 months). They postulated that MLC and its equivalent could predict disease progression to AML [57].

**Figure 4:**
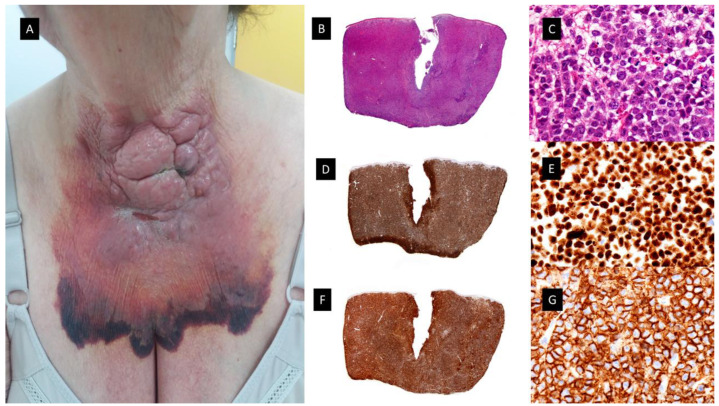
Granulocytic sarcoma. (**A**) Clinical picture showing a large, hemorrhagic, violaceous polilobulated tumor on the anterior neck. (**B**,**C**) Hematoxylin-eosin-stained specimen. (**B**) Scanning view showing diffuse dermal infiltrate (4×). (**C**) Higher magnification with medium-sized monomorphous atypical cells (40×). (**D**,**E**) MNDA-stained specimen [(**D**) (4×) (**E**) (40×)]. (**F**,**G**) CD56-stained specimen [(**F**) (4×), (**G**) (40×)].

Lately, in the study previously mentioned by Vitte et al., the authors recognized four main subtypes of cutaneous involvement by CMML, one of them, as previously discussed, being mature PDC tumors. The most frequent group was myelomonocytic cell tumors (MMCT) (n = 18), exhibiting a proliferation of granulocytic or monocytic blast cells, which were CD68 and/or MPO positive, but negative for dendritic cell markers, similar to what it is known classically as granulocytic sarcoma [45]. According to the criteria of Vitte et al., nine cases from Mathew et al.’s series were diagnosed as MMCT. The median age in this group was 71.5 years with a male to female ratio of 5:1. The lesions were most often papulonodular and multiple. The skin tumors consisted of a proliferation of medium-sized and/or large-sized cells, mainly or entirely comprising blast cells. A reactive infiltrate was present in 70% of the cases, predominantly lymphocytes. Tumor cells displayed a myelomonocytic phenotype, expressing CD68 (17/18), MPO (13/18), CD33 (13/18) and CD13 (4/18). PDC markers were negative in tumor cells. In addition, all cases were negative for CD1a and Langerin, and all but one case were also negative for S100 protein. Ten cases were CD4 positive and five were CD56 positive. Four cases (22%) were both CD4 and CD56 positive. The proliferation index with Mib-1 antibody was highly variable and generally low. The OS was worse than in the group of mature PDC tumors, but better than the other two groups [45]. Taking all these findings together and including the series of Mathew et al. and Vitte et al., these MMCT could be considered as the most frequent form of MLC in CMML patients. (see Figure 4).

#### 3.1.2. Aleukemic LC

Another interesting concept worth discussing when talking about LC is aleukemic LC, which has also been described in MDS/MPN [58,59,60]. It is characterized by the infiltration of the skin by leukemic cells before their appearance in peripheral blood or BM. Their prompt recognition with a high suspect index is of crucial relevance, in order to make a correct diagnosis and distinguish it from other entities such as cutaneous large-cell lymphoma. The prognosis is also poor, as in common MLC [58,59,60]. Cutaneous lesions initially suggestive of ND may actually represent LC, but it must not be confused with myelodysplasia cutis, previously discussed within neutrophilic dermatoses, because the evolution, clinical and histopathological characteristics are different, and having MLC results in a much worse prognosis [30]. Some authors have coined the term “aleukemic cutaneous myeloid sarcoma” or “aleukemic granulocytic sarcoma” when there is an isolated atypical cutaneous infiltrate, reserving the term “aleukemic LC” for multiple skin lesions [61], but there are authors which use both terms interchangeably [62].

### 3.2. Blastic Plasmacytoid Dendritic Cell Tumor (see Figure 5)

This category of cutaneous disorder related to MDS/MPN and aggressive clinical behavior includes the third group mentioned by Vitte et al. in their article as blastic PDC tumors (BPDCT). They included in this group 9.5% of the patients of their study (n = 4). The median age of the patients was 71.5 years, with a male to female ratio of 3:1. Skin lesions were multiple and disseminated in three cases and solitary in one case. They comprised infiltrated plaques or violaceous nodules. Histopathologically, these lesions showed dermal infiltrates of medium-sized blast cells with slightly irregular nuclei and small nucleoli. Immunohistochemical studies revealed that CD68 was positive in three cases, whereas MPO, CD13, and CD33 were all negative. The four cases were CD4 positive, but only three cases showed positivity for CD56. All cases were CD1a negative, but one was S100 protein positive. The four cases were positive for the PDC markers CD123 and TCL1. Two cases were also positive for the PDC marker CD303. All four cases were negative for granzyme B [45]. In 2006, after the recognition of BPDCN as a separate entity, Dijkman et al. carried out an integrated genomic analysis using expression profiling and array-based comparative genomic hybridization (CGH) on the lesional skin biopsy samples of patients with CD4+CD56+ BPDCN and cutaneous myelomonocytic leukemia [63]. Their results demonstrated that CD4+CD56+ BPDCN and cutaneous myelomonocytic leukemia show distinct gene-expression profiles and distinct patterns of chromosomal aberrations. CD4+CD56+ BPDCN and cutaneous myelomonocytic leukemia split into two distinct groups consisting of a group of five CD4+CD56+ BPDCN and a group of three cutaneous myelomonocytic leukemia. CD4+CD56+BPDCN is characterized by recurrent deletion of regions on chromosome 4 (4q34), chromosome 9 (9p13-p11 and 9q12-q34), and chromosome 13 (13q12-q31) that contain several tumor suppressor genes with diminished expression (Rb1, LATS2). Elevated expression of the oncogenes HES6, RUNX2 and FLT3 was found, but it was not associated with genomic amplification. They found high expression of various plasmacytoid dendritic cell (pDC)-related genes in BPDCN, pointing to the cell of origin of this malignancy [63]. 

Recently, Khanlari et al. have published a study showing that bone marrow clonal hematopoiesis (BMCH) is highly prevalent in BPDCN and frequently shares a clonal origin in elderly patients. They assessed a potential clonal relationship between BPDCN and BMHC by comparing mutation profiles. A clonal association of BPDCN with BMCH was confirmed in 13 of their patients (54%) by demonstrating shared common mutations, with *TET2*, *ASXL1* and *ZRSR2* being the most common ones. Although about 30–40% carried a diagnosis of MDS, CMML or MPN, many patients had no clinical manifestations of a myeloid neoplasm. In around 50–60% of cases, BPDCN shared a clonal origin with these pre-leukemic clones but with additional molecular cytogenetic abnormalities [64]. 

Case reports on the common clonal origins of BPDCN and MDS are rare. Recently, Yamada et al. have reported a patient diagnosed with BPDCN limited to the skin and MDS with increased blasts 1 (MDS-IB1) in the BM [65]. They performed targeted NGS and copy number analyses (CNA) that revealed that both originated from the same clonal origin which subsequently evolved into BPDCN by acquiring multiple CNVs. The higher VAFs of the common mutations compared to the other mutations suggest that the genetic events occurred in the early phase and could have originated from CHIP [65]. 

### 3.3. Histiocytoid Disorders/Histiocytosis

Histiocytosis disorders are uncommon disorders of children and adults characterized by the neoplastic proliferation of dendritic cells, macrophages or monocyte-derived cells in various tissues and organs, including the skin. There have been more than 100 different subtypes described in the literature, with a revised classification based on histopathology, immunophenotype, molecular alterations, and clinical and imaging characteristics published in 2016 [66]. 

The last group included by Vitte et al. in their study, which was the less frequent and the one with a worse prognosis, included histiocytic proliferations. They mentioned it as a putatively novel category of tumor that they named blastic indeterminate dendritic cell tumors (BIDCT) (n = 4), distinguished by a proliferation of large blast cells that not only exhibit monocytic markers but also the dendritic markers, CD1a and S100 protein. The median age of the patients in this group was 65.5 years with a male to female ratio of 1:1. Tumors comprised a dermal infiltration of large blast cells with round, oval or irregular nuclei and several nucleoli. Immunohistochemically, the four cases were positive for CD68, CD13, CD33 and CD1a. Three cases were positive for S100 protein, and none of the cases stained for langerin. Two cases were CD4 positive, and three cases were also CD56 positive. Interestingly, one case (case 42) also expressed the PDC marker CD303, whereas all other PDC markers were negative in the four cases. Mib-1 was available for three cases. In two of them (67%), the Mib-1 index was high, being over 66%. The clinical characteristics of skin lesions consisted of a single nodule in two cases, multiple nodules in one case, and a large and ulcerated tumor of the scalp in one case [45]. 

#### 3.3.1. Histiocytic Sarcoma

**Figure 6:**
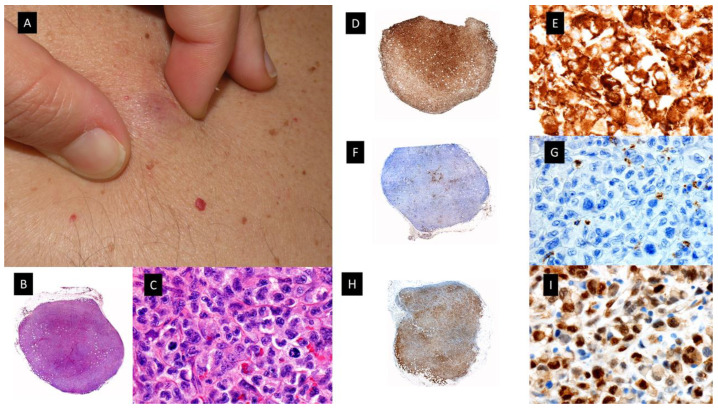
Histiocytic sarcoma. (**A**) Clinical picture showing a subcutaneous nodule of 4 cm on the chest. (**B**,**C**), Hematoxylin-eosin-stained specimen. (**B**) Scanning view of diffuse subcutaneous nodular infiltrate (4×) (**C**) Higher magnification with atypical large cells with eosinophilic cytoplasm (40×). (**D**,**E**) CD163-stained specimen showing diffuse positivity [(**D**) (4×) (**E**) (40×)]. (**F**,**G**) MPO-stained specimen showing negative results [(**F**) (4×), (**G**) (40×)]. (**H**,**I**) p-ERK-stained specimen showing diffuse positivity [(**H**) (4×) (**I**) (40×)].

Histiocytic sarcoma (HS) is an extremely rare histiocytosis with an aggressive course and limited treatment options. Clinically, the disease usually presents with single or multiple extranodal tumors, frequently located in the skin, intestines or soft tissues. The histopathology shows large atypical cells with eosinophilic cytoplasm and immunopositivity to CD163, CD68 and lysozyme. (see Figure 6) The overlap with some forms of non-Hodgkin´s lymphomas (NHL) has resulted in controversy about this diagnosis, and in this regard, a number of earlier cases describing HS are most likely forms of high-grade NHL [67]. In the literature, there have been published cases of HS as a secondary event following the diagnoses of other malignancies, including acute monocyte leukemia [68] and CMML [69]. In addition, Pérez-Sáenz et al. published the findings regarding a patient who initially had CMML and HS on the skin (two nodules on the chest), who subsequently developed a therapy-related AML with monoblastic differentiation. They reported mutations on *KRAS, TET2* and *SRSF2* shared by the three neoplasms, suggesting a possible common clonal origin. The HS and AML showed additional molecular features [70].

#### 3.3.2. Other Histiocytosis

A recently published multi-institutional cohort of 189 patients with Erdheim–Chester disease (ECD) and ECD overlapping with Langerhans cell histiocytosis [so-called mixed histiocytosis, (MH)] showed that 10.1% of patients (19/189) with ECD have an overlapping myeloid neoplasm, most commonly occurring as MPN, MDS or mixed MDS/MPN overlap syndrome (including CMML) [71]. Molecular analysis frequently detected hallmark driver mutations typical of myeloid neoplasms (such as *JAK2V617F* and *CALR* mutations) coexisting with those characteristic of histiocytosis (such as *BRAFV600E* and *MAP2K1* mutations). In addition, histiocytosis patients diagnosed with a concomitant myeloid malignancy were significantly older at diagnosis and more commonly presented with MH than those without a myeloid malignancy. The authors postulated that the presence of distinct kinase mutations in the histiocytosis and myeloid neoplasm resulted in discordant and adverse responses to kinase-directed targeted therapies [71]. 

Later, a Dutch retrospective population-based study identified three patients with different histiocytic neoplasms and additional hematological malignancies bearing identical oncogenic mutations [72]. One patient had concomitant *KRAS* p.A59E mutated histiocytic sarcoma and CMML, one patient had synchronous *NRAS* p.G12V mutated indeterminate cell histiocytosis and CMML and finally, one patient had subsequent *NRAS* p.Q61R mutated ECD and AML [72].

Furthermore, other case reports and series have published the association of cutaneous xanthomatous tumors [73] and xanthelasma-like lesions fulfilling clinical, histopathological and molecular criteria for ECD associated with CMML, with a proven clonal relationship between skin lesions and CMML cells based on molecular analyses using NGS techniques [74], testicular Rosai–Dorfman disease associated with CMML sharing a *KRAS* variant c 0.35 G>A/p.G12D in both [75] and also Langerhans cell histiocytosis (LCH) associated with MDS and other hematological neoplasms [76,77]. There have been some publications which pointed out that the cell of origin of one third of patients with systemic histiocytosis resides in a CD34+ hematopoietic progenitor cell prior to committed monocytes/macrophages [78,79]. Taking this into account, two main biological mechanisms could explain the link between CMML and ECD. On one hand, the first one would be a differentiation from a clonal CMML cell into foamy macrophages in the skin through an additional genetic hit or a transforming event. On the other hand, the second would be a mutant common progenitor cell giving rise to the two distinct neoplasms via divergent differentiation.

In conclusion, these abovementioned studies highlight the clinical importance of evaluating adults with histiocytosis for a concomitant myeloid neoplasm.

## 4. Other Cutaneous Disorders in MDS/MPN Patients

### 4.1. Cutaneous Manifestations Secundary to Microcirculation Abnormalities

Pernio and chilblain lupus in the setting of myeloid neoplasms, especially CMML, are well-known cutaneous disorders described for the first time in four elderly patients with a hematologic disorder whose clinical features were identifiable as CMML [80]. The occurrence of florid pernio in an elderly man with CMML is unusual. In the reported cases, pernio usually preceded the diagnosis of the hematologic disorder by a period of between 6 months and 3 years [81]. The histopathological features of these lesions consisted of a superficial and deep lymphohistiocytic dermal infiltrate associated with lymphocytic vasculitis. Some authors postulated three possible mechanisms for these lesions: (1) The presence of malignant monocytic cells, because of their rigidity, large diameter and difficulty in passing through the vascular lumen may cause modifications in vascular flow in the microcirculation; (2) The polyclonal activation of B lymphocytes with polyclonal hypergammaglobulinemia with autoantibodies secretion; and finally (3) The role of cold, which has an influence on blood viscosity [82]. The immunohistochemical demonstration of the lymphoid nature of the infiltrate and ruling out specific leukemic infiltrates allows the histopathological differential diagnosis of the lesions of chilblain-like leukemia cutis described before [83]. 

Other chronic myeloproliferative disorders associated with livedoid and purpuric cutaneous lesions due to microcirculation abnormalities are essential thrombocythemia [84,85]. Due to the elevated platelet count, the main histopathologic finding in these lesions consists of luminal obliteration by homogeneous eosinophilic material of medium-sized blood vessels. Immunohistochemistry demonstrates positivity for the platelet marker, CD61, in the eosinophilic material, without features of vasculitis, characteristic of occlusive vasculopathy [84,85].

### 4.2. Other Inflammatory/Autoimmune Diseases

There are still some other cutaneous manifestations more rarely reported, which cannot be included in one of the previously described categories. The most important are the ones related to autoimmunity. Autoimmune paraneoplastic syndromes are commonly encountered in patients with MDS and CMML. A review of case reports and small series suggest as many as 10% of MDS patients might experience several autoimmune syndromes. Clinical manifestations of these syndromes include an acute systemic vasculitic syndrome, cutaneous vasculitis, fever, arthritis, pulmonary infiltrates, peripheral polyneuropathy, inflammatory bowel disease, glomerulonephritis, and even classical connective tissue disorders, such as relapsing polychondritis, posing sometimes a diagnostic challenge with VEXAS syndrome [86]. 

Other very rare reported cutaneous lesions related to these myeloid neoplasms are the ones derived from extramedullary hematopoiesis [87].

## 5. Conclusions

Interestingly, in recent literature there are increasing numbers of papers suggesting that the skin infiltrates of dermatoses that arise in the context of myeloid diseases, particularly H-SS, MDS-cutis, PNGD and plasmacytoid cell dendritic cell dermatoses, carry the same molecular alterations as the malignant clone found by FISH, pyrosequencing or next-generation sequencing techniques. Moreover, this relationship has also been established for histiocytosis. 

In conclusion, to date, the cause of the appearance of this wide range of clinical and histopathologic skin lesions in patients with MDS/MPN and its biological meaning is not known. In some cases, cutaneous manifestations, like granulomatous dermatoses, could represent an additional criterion for the early diagnosis of CMML/MDS. These studies suggest a high degree of plasticity present in myeloid, monocytic and PDC cells that can originate from the same precursors and this review article summarizes the cutaneous manifestations of these rare myeloid neoplasms, highlighting the importance that they have in the diagnosis and management of these challenging patients.

## Figures and Tables

**Figure 5:**
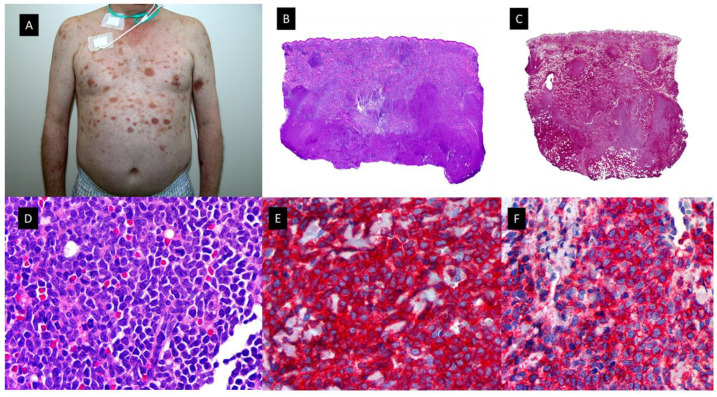
Plasmacytoid dendritic cell tumor. (**A**) Multiple bruise-like plaques on the trunk and upper extremities. (**B**) Scanning view showing diffuse tumoral infiltrate in dermis and subcutaneous tissue (HE staining 4×) (**C**) CD4 positive diffuse staining in the tumoral infiltrate (×4). (**D**) Higher magnification showing monomorphic proliferation of medium-sized blastoid cells and intratumoral hemorrhage (HE staining ×40). (**E**) Detail of the positivity of the majority of neoplastic cells for CD123 (40×) (**F**) Tumoral cells showing also positivity for the cytoplasmic marker CD56 (40×).

## Supplementary Materials

The following supporting information can be downloaded at: https://www.mdpi.com/article/10.3390/cancers15245888/s1, Table S1: MDS/MPN related dermatoses with indolent clinical course with main clinical, epidemiological, immunophenotypic and genetic features; Table S2: Cutaneous processes related to either MDS/MPN and aggressive clinical behavior with main with main clinical, epidemiological, immunophenotypic and genetic features. References [6,7,12,13,15,16,24,25,29,30,35,37,38,39,45,46,47,51,52,53,56,57,58,64,65,66,67,68,69,70,71,72,73,75,77,79] are cited in the supplementary materials.
