# Supplementary material for: Clinical, Histopathological and Molecular Spectrum of Cutaneous Lesions in Myelodysplastic Syndrome and Myeloproliferative Neoplasms (MDS/MPN): An Integrative Review
Source: Cancers (Basel). 2023 Dec 18;15(24):5888. doi: 10.3390/cancers15245888 (PMC10742063; doi:10.3390/cancers15245888)
Supplement: Supplementary file 1 [file cancers-15-05888-s001.zip › cancers-2739201-supplementary.pdf]

Table S1. MDS/MPN related dermatoses with indolent clinical course with main clinical, epidemiological, immunophenotypic and genetic features. NGS: Next Generation Sequencing, MDS/MPN: Myelodysplastic/Myeloproliferative, MPO: Myeloperoxidase MDS-cutis: Myelodysplasia cutis, CMML: Chronic myelomonocytic leukemia, MM: Multiple Myeloma, BM: Bone marrow * Further research is needed to establish the true prevalence of these dermatoses in MDS/MPN.

MDS/MPN DERMATOSES WITH INDOLENT CLINICAL BEHAVIOUR	EPIDEMIOLOGY*	CLINICAL PRESENTATION	HISTOLOPATHOLOGICAL FEATURES	INMUNOPHENOTYPE	MOLECULAR FINDINGS	EVOLUTION	MOST RELEVANT PUBLICATIONS
HISTIOCYTOID SWEET SYNDROME	30 to 61% association with malignancy reported. Most frequent associated neoplasm is MDS	Fever and neutrophilic leukocytosis with erythematous tender cutaneous plaques	Intense superficial dermal edema and underlying band-like dermal infiltrate composed of immature myeloid cells	CD15+, CD43+, CD45+, Lysozyme+, MPO+<br><br>CD68/KP1+>CD68/PGM1+, MNDA+ (Double IS MNDA/MPO+)	Not well-defined yet	Good response to oral steroids	Martin de Frémont et al,2021 [6]<br>Requena et al, 2005 [12]<br>Alegría et al, 2017 [13]<br>Ghoufi L et al, 2016 [15]
VEXAS SYNDROME	Rare disease, 1 in every 13591 adults	Adult-onset autoinflammatory syndrome with overlapping features with MDS, MM, relapsing polychondritis polyarteritis nodosa or giant-cell arteritis.<br>Neutrophilic dermatoses similar to MDS-cutis	Neutrophilic infiltrate in the upper dermis with edema, red cell extravasation and limited leukocytoclasis. Some cases show vasculitis in deep dermal vessels.	MPO+, MNDA+	Somatic mutations in *UBA1* gene located in X chromosome	Poor response to conventional therapies and frequent relapses	Beck DB et al, 2020 [24]<br>Beck DB et al,2023 [25]<br>Nguyen JK et al,2022 [29]
MDS-CUTIS	Not well-studied yet<br>Osio et al examined skin biopsies in 800 patients with MDS and found 24 patients with MDS-cutis	Diffuse papulonodules similar to myeloid sarcoma/leukemia cutis lesions with a relapsing/remitting course in same patients.	May have papillary dermal edema Immature myeloid cells which sometimes show "pince-nez" or pseudo- pelger-Huet anomaly	CD33+, CD163+, CD14+, CD68/PGM1+, MPO+(Double IS CD123+/MNDA+)<br>CD34- CD117-, CD56-, low ki67<br>CD3+ lymphoctyes	NGS techniques have found matching mutations of at least 1 gene in skin and BM of patients with MDS/MPN	Patients may have a more aggressive course than seen in MDS, respond better to hypomethylating agents than to steroids	Whittington CP et al,2023 [7]<br>Delaleu J et al, 2022 [16]<br>Osio A et al,2015 [30]
GRANULOMATOUS DERMATOSES	Only case reports and case series published	Several months duration of 1–2 mm confluent papules coalescing in plaques on the trunk and extremities, sometimes preceding the diagnosis of the hematological disease	Superficial granulomatous dermatitis, with mixed inflammatory elements, such as lymphocytes, scattered neutrophils, and multinucleated giant cells	CD14+, CD68/PGM1+, CD123+<br>CD56-, S100-, CD1a-	NGS techniques and pyrosequencing have found matching mutations of at least 1 gene in skin and BM of patients with MDS/MPN, the most frequent is the *SRSF2* gene mutation	Poor response to conventional therapies, better response to hypomethylating agents than to steroids similar	Yoneka K et al,2016 [35]<br>Prieto-Torres et al,2022 [46]<br>Federmann B et al,2017 [37]<br>Kyriakou A et al,2019 [38]<br>Enescu CD et al,2022 [39]
MATURE PLASMACYTOID DENDRITIC CELL DERMATOSES	Reported in 16 of 42 patients with CMML and skin lesions studied by Vitte et al and other isolated case reports and small series	More heterogenous than the others, including disseminated erythematous macules and papules, nodules similar to insect-bites and vasculitis-like rash	Dermal infiltrates of mature T-lymphocytes with large clusters of PDC in which focal epidermotropism and vacuolar degeneration of dermo-epidermal interface but without mucin deposition can be found.	Double IS CD123+/SPIB + CD56-, CD34-, CD117-, MPO-, TDT-, Bcl2-, MNDA-	NGS techniques and pyrosequencing have found matching mutations of at least 1 gene in skin and BM of patients with MDS/MPN	Cutaneous lesions disappeared spontaneously or after topical or oral treatment with steroids. In all cases published by Machan et al, the skin lesions developed coincidentally with either progression of full-establishment of their hematological condition	Vitte F et al, 2012 [45]<br>Machan S et al, 2022 [46]<br>Dargent JL et al, 2016 [47]
CHILDBLAIN-LIKE ERUPTIONS	Only case reports and case series published	Bluish erythematous macules with mild itching on the toes of both feet	Dense superficial perivascular dermal infiltrate	CD68+, CD14+/-<br>CD13-, CD33-, CD34-, CD56-	Not known	It can be the first manifestation of a blast crisis or a relapse of AL being an important clue	Brazao C et al,2023 [51]<br>Yazawa H et al,2004 [52]<br>Affleck AG et al, 2007 [53]

Table S2. Cutaneous processes related to either MDS/MPN and aggressive clinical behavior with main with main clinical, epidemiological, immunophenotypic and genetic features. AML: Acute Myeloid leukemia, NGS: Next Generation Sequencing, MDS/MPN: Myelodysplastic/Myeloproliferative, MPO: Myeloperoxidase, ECD Erdheim-Chester disease, LCH: Langerhans cell histiocytoses.

MDS/MPN ASSOCIATED PROCESSES WITH AGGRESSIVE CLINICAL BEHAVIOUR	EPIDEMIOLOGY*	CLINICAL PRESENTATION	HISTOLOPATHOLOGICAL FEATURES	INMUNOPHENOTYPE	MOLECULAR FINDINGS	EVOLUTION	MOST RELEVANT PUBLICATIONS
LEUKEMIA CUTIS/MYELOID LEUKEMIA CUTIS	2,1-30% depending on the underlying form of leukemia<br>Its prevalence can reach 50% in some series of AML of monocytic subtypes	Persistent papulonodule(s) and or tumors<br>+/- circulating blasts<br>+/- anemia and thrombocytopenia	Proliferation of medium-sized and/or large sized cells, mainly or entirely blast cells. A reactive infiltrate was present in 70% of the cases, predominantly lymphocytes	CD68+, MPO+, CD33+, CD13+, high ki67<br>More likely to express CD34, CD117	Mutations identified in most cases by NGS	Poor prognosis	Vitte F et al, 2012 [45]<br>Mathew et al, 2012 [58]<br>Cho-Vega JH, 2008 [56]<br>Kaddu S, 1999 [57]
BLASTIC PLASMACYTOID DENDRITIC CELL TUMOR	Very rare<br>9.5% of cases in the study published by Vitte et al	Bruise-like infiltrated plaques or violaceous nodules	Diffuse tumoral infiltrate in dermis and subcutaneous tissue composed by monomorphic proliferation of medium-sized blastoid cells +/- intratumoral hemorrhage	CD4+, CD56+ high Ki67, SPIB+, CD123+<br>MPO-, CD13-, CD33-, MNDA-	Recurrent deletions of regions on chromosomes 4,9 and 13 (diminished expression of *Rb1, LATS2*).<br>Elevated expression of oncogenes *HES6, RUNX2, FLT3*.<br>Shared common mutations with myeloid neoplasms, being *TET2, ASXL1* and *ZRSR2* the most common ones	Poor prognosis	Vitte F et al, 2012 [45]<br>Dijkman R et al, 2006 [64]<br>Khanlari M et al, 2022 [65]<br>Yamada T et al, 2023 [66]
HISTIOCYTOID DISORDERS/HISTIOCYTOSES	A recent study showed that 10,1% of patients with ECD have an overlapping myeloid neoplasm	HISTIOCYTIC SARCOMA: single or multiple extranodal tumors, frequently located in the skin, intestines or soft tissues.<br>ECD: Xanthelasma-like cutaneous lesions, nonspecific patches or papulonodular lesions, predominantly affecting the legs, back, and/or trunk, may also be observed and other systemic manifestations (bone, cardiovascular, CNS, pulmonary, endocrine..)<br>LCH: Erythematous papules involving trunk, head and extremities, especially seborrheic areas.	HISTIOCYTIC SARCOMA: Large atypical cells with eosinophilic cytoplasm<br>ECD: Infiltration of tissues by foamy mononucleated histiocytes with small nucleus. A few multinucleated histiocytes or Touton cells are also frequently observed. Fibrosis and reactive lymphocytes, plasma cells and neutrophils are also frequent.<br>LCH: In the dermis and focally in the epidermis there is a proliferation of cells with reniform nuclei and abundant eosinophilic cytoplasm.	HISTIOCYTIC SARCOMA: CD163+, CD68+, Lysozyme+, pERK+, MPO-<br>OTHERS:<br>ECD:CD68+, CD163+, CD1a-<br>LCH: S100+, CD1a+, Langerin/CD203+	HISTIOCYTIC SARCOMA: mutations on KRAS, TET2 and SRSF2<br>OTHERS: Hallmark driver mutations typical of myeloid neoplasms (such as JAK2V617F and CALR mutations) coexisting with BRAFV600E and MAP2K1 mutations.	Poor prognosis	Vitte et al, 2012 [45]<br>Emile JF et al, 2016 [67]<br>Ansari J et al, 2016 [68]<br>Zhao J et al, 2015 [69]<br>Mori M et al, 2010 [70]<br>Pérez-Saenz MA et al, 2020 [71]<br>Papo M et al, 2017 [72]<br>Kemps PG et al, 2021 [73]<br>Bonnet P et al, 2019 [75]<br>Kiavash K et al, 2018 [77]<br>Durham BH et al, 2017 [79]
